# Practical Applications of Lung and Diaphragm Ultrasound in the Intensive Care Unit: An Updated Narrative Review

**DOI:** 10.7759/cureus.88584

**Published:** 2025-07-23

**Authors:** Keith Killu, Monika Kakol

**Affiliations:** 1 Department of Medicine, University of Southern California Keck School of Medicine, Los Angeles, USA

**Keywords:** acute respiratory distress syndrome (ards), a-lines, b-lines, diaphragm ultrasound (d-usg), lung ultrasound (lus), percutaneous tracheostomy, pleural fluid (pf), pleural ultrasound, pneumonia, point-of-care ultrasound (pocus)

## Abstract

Lung ultrasound (LUS) has evolved significantly during the past few decades. Its use has been integrated into daily practices of intensive care units (ICUs) worldwide, and it has proven to be a valuable tool in the assessment and management of patients with respiratory failure caused by lung, pleural, diaphragmatic, and other diseases. LUS techniques are becoming increasingly standardized, which can help in interpreting data and improving patients’ outcomes. In this narrative review, the focus was on the practical daily applications of lung, pleural, and diaphragmatic ultrasound with emphasis on different signs and artifacts that guide the interpretation of data and identification of disease. Discussions and analysis from the new international guidelines were added to help close the gap in the use of LUS. This review is intended to serve as a practical guide for using bedside ultrasound in evaluating patients with shortness of breath and respiratory failure and to provide guidance to help providers manage patients and generate standardized reports. We start with an analysis of best practices and guidelines on performing an LUS exam in the ICU setting. This analysis is followed by data interpretation of findings starting at the pleural line and traveling deeper into the lung tissue. The review includes discussions of the diaphragm evaluation and its function and abnormalities, as well as common LUS-related procedures in the ICU, such as thoracentesis, tracheostomy, and cricothyrotomy.

## Introduction and background

Using ultrasound to examine the lungs was once considered a suboptimal practice owing to the poor conductivity for ultrasound waves in air-filled lung tissue. This understanding was initially accepted and not widely challenged, but as technologies have advanced over the past 50 years, lung ultrasound (LUS) applications have significantly progressed. In 1964, Pell [1] published one of the earliest studies on LUS and its clinical applications, describing a diagnosis of right-sided pleural effusion by ultrasound and the follow-up results after thoracentesis. He reported that ultrasound does not penetrate healthy aerated lung tissue very well and the echo is rapidly attenuated, but pleural fluid is a good conductor of ultrasound. In the early 1990s, most of the initial signs and artifacts were recognized through research [2]. In particular, use of LUS to assess the surface structures and the pleural line can yield specific signs that help distinguish normal and disease conditions. For deeper structures such as the lung parenchyma that have a significant interface between ultrasound and air, the artifacts observed in LUS results can be categorized as indicative of normal or disease conditions. Most lung parenchymal pathologies (especially consolidations) reach the surface at the pleural line, producing specific signs that can be easily detected by ultrasound.

This review covers the steps in performing LUS examination and the interpretation of signs and artifacts, starting from the pleural line and moving to deeper structures and the diaphragm. It includes the most recent advances, including the application of artificial intelligence (AI) as an aid in interpreting data.

## Review

Performing LUS and the technical aspects

Various professional societies and experts gathered at the first international LUS conference to create a consensus statement on LUS in 2012 [3]. In 2023, new international guidelines were published [4] with more detailed recommendations about the performance and interpretations of LUS and a roadmap for future research. The following sections describe important considerations and steps for patient examinations and machine preparation.

Getting Started

A focused and productive LUS examination requires identifying why it is needed before it is started. For example, the examination may be necessary for uncovering the specific reason that a patient is presenting symptoms of respiratory failure or for determining why weaning a patient from mechanical ventilation has failed.

Examination in the Intensive Care Unit (ICU) Setting

LUS has unique characteristics and limitations when it is being done on critically ill ICU patients. Most patients are in a supine or semi-recumbent position, and they are usually sedated and possibly hemodynamically unstable. These factors make LUS examination difficult and might limit its application.

Choosing the Transducer Type and Scanner

The transducer choice is important in identifying different structures, signs, and artifacts. Using lower frequency transducers is better when trying to interpret deeper reflections. The curvilinear (2-5 MHz) and phased array (2.5-5 MHz) transducers are used for this purpose and specifically for identifying lung parenchymal tissue abnormalities. For examination of the pleural surface, a higher frequency transducer with better resolution is needed, and a linear transducer (7-14 MHz) is used. Familiarity with the ultrasound machine and its functionality is necessary. Furthermore, using the same equipment helps decrease image variability and data inconsistency in most cases, and it can probably reduce training inconsistencies.

Areas of Chest Examined

**Figure 1:**
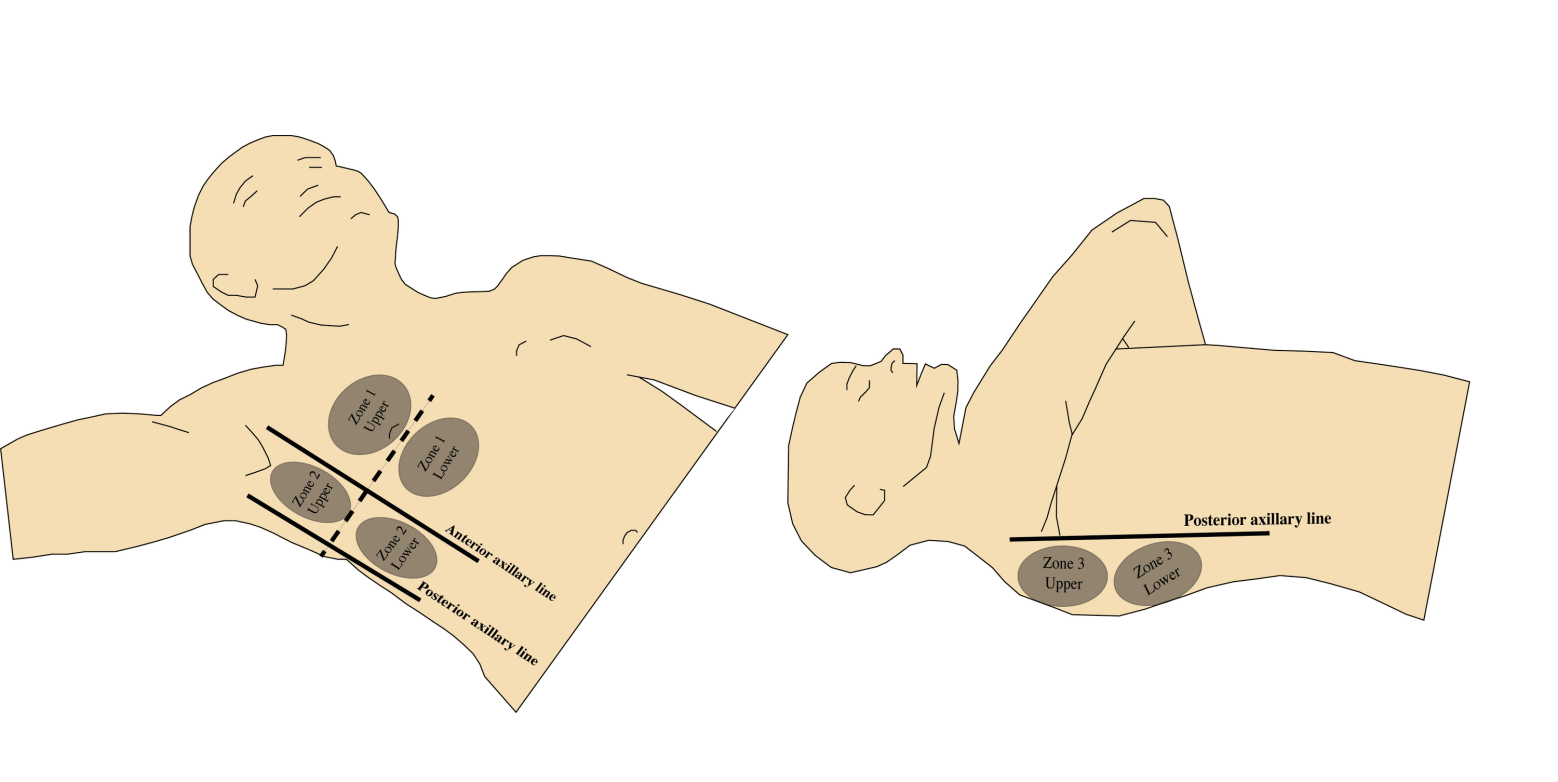
Lung zones. Zone 1: upper and lower anterior; Zone 2: upper and lower lateral; Zone 3: upper and lower posterior. Credit: Image created by the authors.

Most literature divides lung zones into upper and lower areas in the anterior chest wall (between the midline and the anterior axillary line), upper and lower lateral chest wall (between the anterior and the posterior axillary lines), and upper and lower posterior chest wall (between the posterior axillary line and the spine) [2,5,6], which results in a total of six zones on each side (Figure 1). In an ICU patient, the number of zones that can be examined may be limited because of the patient’s supine position [7,8]. Lifting the arm across the chest in a supine patient allows examination of the posterior zones with minimal need to turn the patient. Lung zones included in a practical ICU examination are shown in Figure 1.

Choosing the Total Imaging Depth

Image depth is important for identifying specific artifacts beyond the pleural line or enhancing images at the pleural line. For example, vertical lines (B lines) in the same patient and in the same zone area will differ according to depth, owing to adjusting the focus and gain. The main parameter that can affect the clarity of the vertical lines (B lines) is the frequency of the transducer [9]. Generally, when a linear transducer is used to examine the pleural line, the depth is set at about 3-4 cm. When a phased array or curvilinear transducer is used for deeper structures, the depth is usually set at about 8-10 cm.

Adjusting the Focal Point

Focusing the ultrasound waves at a certain depth of interest leads to better resolution and image quality, and it concentrates the ultrasound beams into a specific area for better lateral resolution. Almost all new ultrasound machines adjust the focal zone automatically. If the machine does not have automatic detection and positioning for the focal point, the operator must adjust it manually.

Adjusting the Gain

Adjusting the gain on the screen affects the brightness of the image and the resolution obtained. Most machines adjust the gain automatically, but some use time gain compensation to help adjust the gain at different levels for different depths. Too much gain leads to very bright images, and too little gain can yield suboptimal images. Optimal gain helps identify structures with better resolution.

Observing the Mechanical Index (MI)

MI is the measure of power of an ultrasound beam. LUS is not absolutely risk-free. It can lead to pulmonary capillary hemorrhage, as shown in animal models, by affecting the alveolar epithelial gas interface and leading to microvascular injury and capillary leak [10]. The MI should be set between 0.4 and 0.7 [4], with the least amount of time of exposure to ensure that the effect on lung tissue is minimal. The Food and Drug Administration has approved an MI value of 1.9 as the maximum threshold for general diagnostic imaging to avoid the bioeffects [4]. In general, whenever using ultrasound, the operator should follow the ALARA (as low as reasonably achievable) principle to minimize the bioeffect of acoustical energy on tissues. If the MI is high, the recommendation is to decrease it if possible or to contact the manufacturing company to adjust.

Chest Wall Thickness

The chest wall thickness is an important consideration because increased subcutaneous tissue and greater chest wall thickness can affect which transducer and frequency are appropriate to use. In addition, reports are needed on any hematomas, deformity, or abnormality present. For a patient with a thin chest wall, minimal subcutaneous tissue, or less muscle mass, a linear transducer is sufficient to evaluate the pleural line, lung parenchyma, and artifacts as the vertical lines (B lines) and horizontal lines (A lines).

Disinfecting the Ultrasound Equipment

Cleaning and disinfecting the ICU ultrasound machine should be a routine practice [11], before and after each use. This practice helps prevent the spread of hospital-acquired infections and follows other infection control recommendations by many societies, helping to decrease complications and rates of infection [12]. Cleaning can be accomplished by soap and water or special chlorine-free wipes to avoid damage to underlying ultrasound crystals in case of a non-intact transducer surface cover.

Rationale for the Choices Made

When assessing an LUS examination report, the reader or reviewer must have information on the reasoning behind choices made. Therefore, it is recommended to provide a summary of all choices mentioned.

Signs and artifacts

Signs refer to ultrasound reflections representing images of actual structures. Artifacts arise when the ultrasound beam reaches an interface where there is a significant amount of air (lungs), and reflections are organized in a certain pattern (e.g., horizontal lines, or A lines). Signs and artifacts are discussed in this section according to their site of origin, starting from the chest wall and extending to the pleura and subpleural structures as shown in Table 1.

**Table 1:** Lung ultrasound artifacts and signs. ARDS: acute respiratory distress syndrome; COPD: chronic obstructive pulmonary disease; ILD: interstitial lung disease; PE: pulmonary embolus

Signs and artifacts	Description	Clinical significance
Bat sign	Formed by two ribs and the pleural line in between	Normal
Lung sliding sign	Movement of the visceral against the parietal pleura	Normal; rules out pneumothorax
Seashore sign	M-mode showing smooth (above) and grainy (below) areas relative to the pleural line	Normal; rules out pneumothorax
Stratosphere sign	M-mode showing smooth (above) and smooth (below) areas relative to the pleural line	Possible pneumothorax
Lung point sign	Localized transition point from the intrapleural air (no lung sliding) to normal pleural movement (lung sliding)	Pneumothorax
Lung pulse sign	Transmission of the heartbeat to the pleural line when the pleural line is not moving	Lack of ventilation, pleural adhesions; rules out pneumothorax
A lines (horizontal lines) artifact	Due to reverberations of the pleural line	Normal aeration; can differentiate hypoxemia due to COPD versus pulmonary edema
B lines (vertical lines) artifact	Due to reverberation possibly from thickened interlobular septa and extravascular fluid (B7 lines) or interstitial and alveolar fluid (B3 lines)	Indicate interstitial alveolar disease, such as pulmonary edema, ARDS
Dynamic air bronchogram sign	Hyperechoic artifacts within the lung parenchyma due to air movement with respiration	Open airways and possible pneumonia
Static air bronchogram sign	Hyperechoic artifacts within the lung parenchyma with no movement with respiration	Closed airways and atelectasis
Consolidations		
Shred sign	Shredded like tissue with a lung line of irregular border. Involves small area (non-trans-lobar) involvement	Parenchymal disease due to pneumonia, interstitial alveolar syndrome, such as ARDS, ILD, PE
Tissue-like pattern sign	Trans-lobar involvement representing as a tissue-like pattern (resembling liver tissue)	Pneumonia

Signs and Artifacts Originating From the Chest Wall

**Figure 2:**
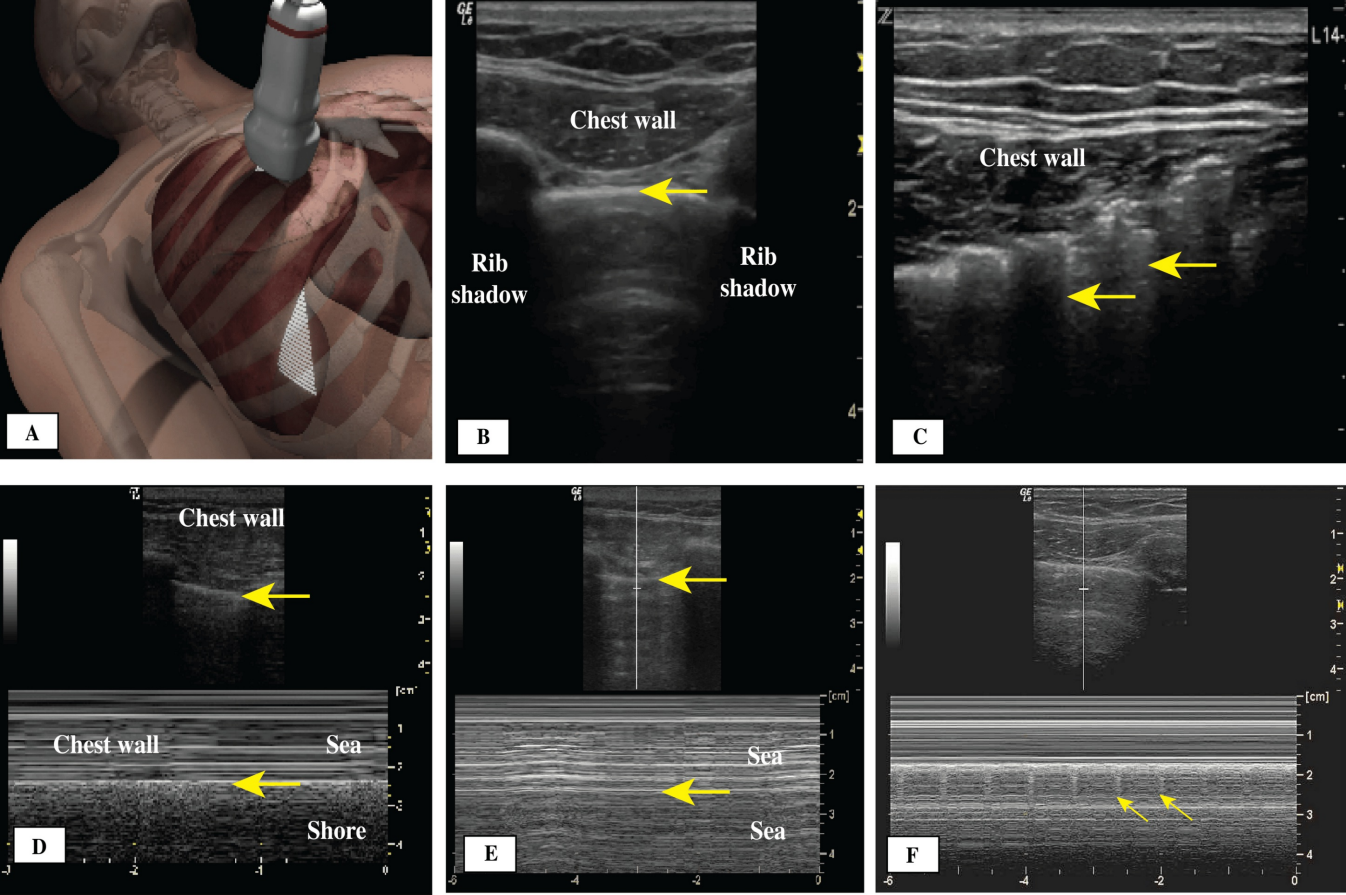
(A) Initial transducer placement location; (B) Bat sign: normal thin pleural line (arrow) between two rib shadows; (C) E lines (arrows); (D) M-mode, seashore sign, pleural line (arrows); (E) M-mode, stratosphere sign, pleural line (arrows); (F) Lung pulse sign showing transmitted heart beats to the pleural line.

At the start of an LUS examination, an initial view should be obtained by placing the transducer perpendicular to the chest wall (Figure 2A), generating what is known as the bat sign, which includes the chest wall, the pleural line, and two ribs (Table 1 and Figure 2B). This initial view enables the operator to become oriented to different structures. When subcutaneous air is present, it obscures most of the structures, including the pleural line, from view and generates artifacts called the E lines sign (Figure 2C). E lines arise from the muscle and subcutaneous layers and not the pleural line, which helps differentiate them from the vertical lines (B lines) that originate from the pleural line. The E lines do not have synchronous movement with respiration, but the vertical lines (B lines) have those movements with respiration. Signs and artifacts originating from the chest wall are shown in Figure 2.

Signs Originating From the Pleural Line

**Figure 3:**
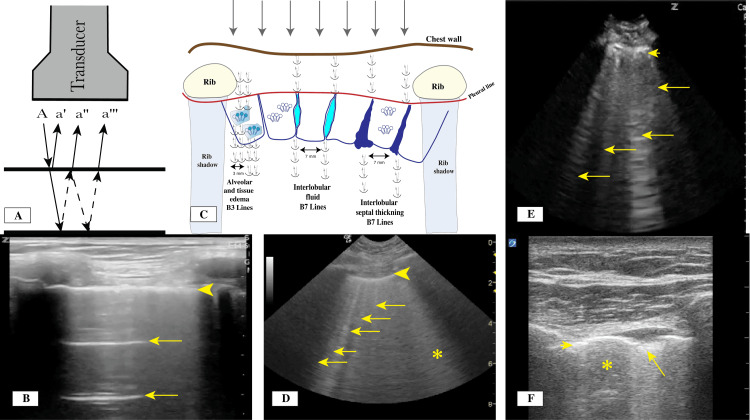
(A) Reverberation artifact mechanism for the horizontal A lines. (B) With the use of a linear transducer, a thin pleural line (arrowhead) and horizontal lines (A lines; arrows) are visible. (C) Proposed mechanism for the reverberation and formation of vertical lines (B lines). (D) With the use of a curvilinear transducer, a thin pleural line (arrowhead) with multiple vertical lines (B3 Lines) with 3 mm between visible lines can be seen in a patient with pulmonary edema. The asterisk indicates white lung with coalesced B Lines. (E) With the use of a curvilinear transducer, a thick pleural line (arrowhead) with multiple vertical lines (B7 lines) (arrows) with wider spaces between the B lines can be seen in a patient with thickening interlobular septa. (F) With the use of a linear transducer, an interrupted thin pleural line (arrowhead), subpleural consolidation (arrow), and skip area (asterisk) are visible. Credit: Images A and C created by the authors.

**Figure 4:**
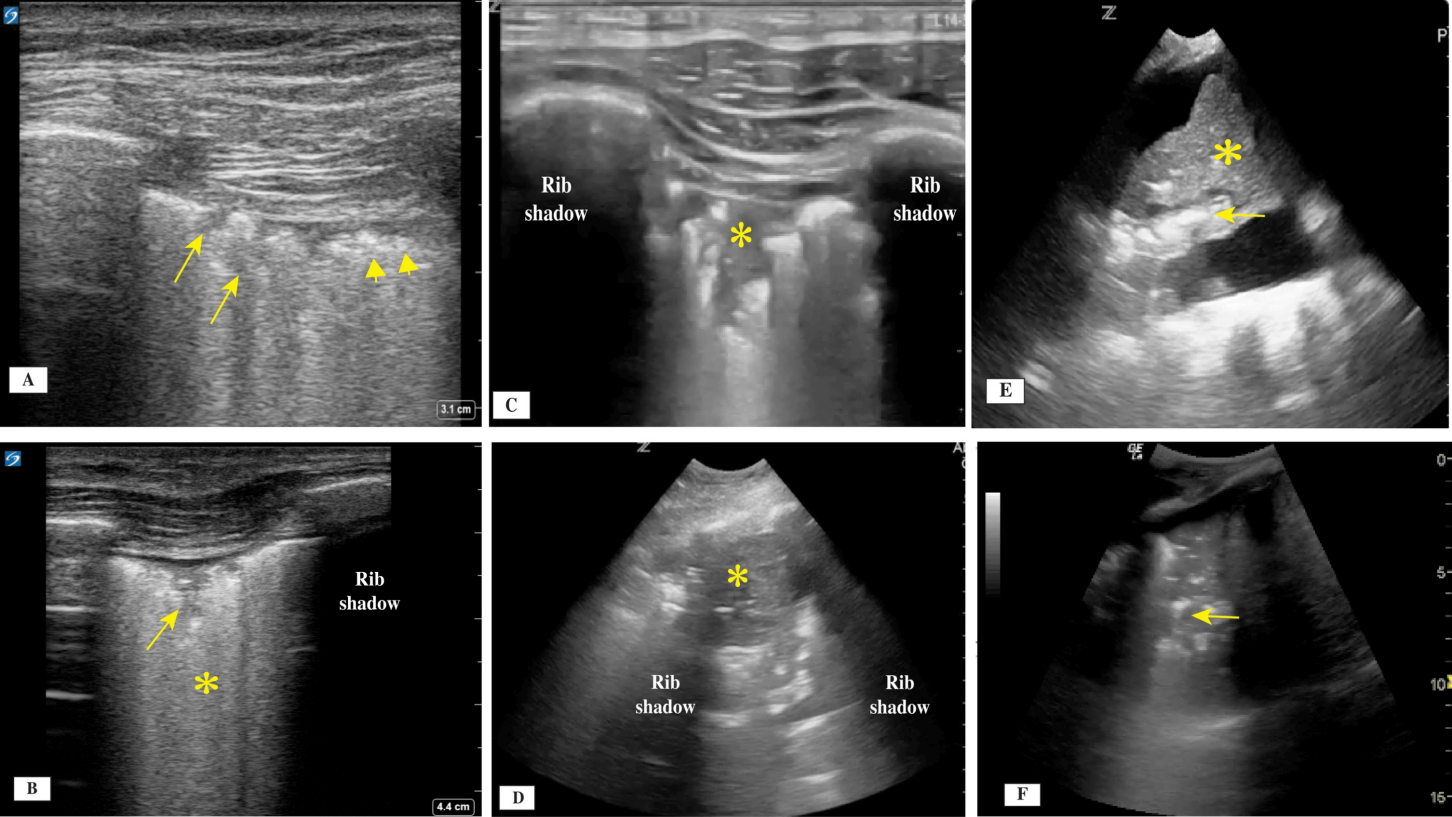
(A) With use of a linear transducer, a thick pleural line (arrowheads) with multiple areas of subpleural consolidations (arrows) could be visualized. (B) A linear transducer also enabled visualizing a thick interrupted pleural line with subpleural consolidation (arrow) and numerous vertical lines forming the white lung sign (asterisk). (C) A linear transducer revealed a shred sign (asterisk) with interruptions and irregularity of the pleural line. (D) With use of a curvilinear transducer, a tissue-like pattern representing trans-lobar consolidation (asterisk) from pneumonia was shown. (E) A curvilinear transducer was used and showed dynamic air bronchogram with longitudinal bright shape (arrow), moving through the airway during the respiratory cycle, and collapsed lung tissue (asterisk). (F) A curvilinear transducer was used and revealed static air bronchogram, which usually resembles small circles that do not move with respiration.

The pleural line can be identified below the chest wall muscle layer. It is normally a thin, bright hyperechoic straight line with no interruptions. Its movement is created by the motion of the parietal and visceral pleura during respiration, generating what is called the lung sliding sign (Figure 2B, Video 1). Closer examination of the pleural line is achieved using a high-resolution linear transducer. The thickness, continuity, and regularity of the pleural line should be described because it can vary significantly in different conditions. A normal pleural line is smooth and less than 2 mm in thickness (Figures 3B, 3D), moves with respiration, and has no interruptions in its continuity and no irregularities of the surface. In patients with acute respiratory distress syndrome (ARDS) and interstitial lung disease (ILD), there will be scattered areas of normal pleural line and multiple areas where the continuity is interrupted, and the line itself could have increased thickness and irregularity (Figures 4A-4C).

**Video 1:** Lung sliding.

Lung sliding is an important sign, and its presence indicates no separation between the visceral and parietal pleura and allows ruling out pneumothorax (Video 1). Applying the M-mode can aid in identifying the moving pleural layers against each other versus no movement at all, depicted by the sea-shore sign and the stratosphere sign, respectively (Figures 2D, 2E). In the mid 1990s, Lichtenstein and Menu [13] described the lung sliding sign and its significance in LUS examinations, especially in ruling out pneumothorax, with 95% sensitivity and 91% specificity. In 2005, Blaivas et al. [14] reported similar results in trauma patients with pneumothorax. In many instances, LUS could be superior to chest X-rays in determining the presence or absence of pneumothorax. A recent meta-analysis found that the use of LUS in supine trauma patients was superior to chest X-rays in the emergency department [15]. Lung sliding is shown in Video 1.

The lung point sign (Video 2) occurs when the pleural line movement is interrupted at the pneumothorax site where the visceral and parietal pleural lines separate from one another, and it is 100% specific for pneumothorax [16]. Lung point location can be used to estimate the size of the pneumothorax. A small pneumothorax is defined by the lung point being found anterior to the mid-axillary line, and a large pneumothorax is identified when the lung point is posterior to the mid-axillary line [17]. Lung point is shown in Video 2.

**Video 2:** Lung point.

The lung pulse sign is also related to the pleural line. It appears when there is complete or near-complete atelectasis of one lung, as in main stem intubation. In such cases, lung sliding is absent on the affected side, and the effect of transmitted heartbeat on the pleural line can be visualized (Figure 2F) [18].

A combination of the absence of lung sliding, vertical lines (B lines), and lung pulse, and the presence of lung point indicates the presence of pneumothorax. A meta-analysis examining the use of LUS in detecting pneumothorax showed an overall sensitivity of 78.6% and specificity of 98.4% [19].

Signs and Artifacts Originating From the Subpleural Space

Horizontal lines (A line artifacts) form owing to the strong reflective property and echogenicity of the pleural line, leading to the development of reverberation artifacts (Figures 3A, 3B). The presence of horizontal lines is considered a normal variant and can point to a high gas-volume (air-lung) ratio below the visceral pleura, as in patients with chronic obstructive pulmonary disease (COPD). These lines are repeated at equal intervals and are equal to the distance between the skin and the pleural line. In the absence of lung sliding and lung pulse signs, the presence of horizontal A lines is very specific for pneumothorax, but their presence along with vertical lines rules out pneumothorax.

Vertical lines (B line artifacts), sometimes called comet tail artifacts [20], can develop owing to reverberations that are thought to be secondary to the thickening or edema in the interlobular septa caused by the accumulation of fibrotic tissue, inflamed tissue, or the development of interstitial edema (Figures 3C-3E). They originate from the pleural line, extend to the bottom of the ultrasound screen, and move with the movement of the pleural line sliding. When present, they usually erase the horizontal lines. They can represent a normal variant, especially when only one or two are present in a zone area. They usually indicate an increase in the lung and interstitial tissue density relative to air. Different artifacts and signs originating from the pleural surface are illustrated in Figure 3.

The pattern of distribution for the B lines is important since it can point toward a certain disease or pathology. A collection of B lines that are separated by about 7 mm (B7 lines) usually indicates thickening or fluid collection between interlobular septa. A smaller distance between the B lines, about 3 mm (B3 lines), usually indicates interstitial or alveolar fluid collection or edema (Figure 3C).

Lung water identification and monitoring can be done using this sign. A more dense accumulation of B lines indicates more severe pulmonary edema and lung water, and the lines decrease with treatment [21,22].

B lines that are scattered with skip areas of normal pleural line (Figure 3F) with no vertical lines in between can indicate ARDS or ILD, rather than pulmonary edema, for which the pattern is continuous without skip areas (Figure 3D). A more focal pattern, called the B pattern, arises when the vertical lines are present in a specific area, which could indicate a pneumonic process, atelectasis, or pulmonary embolism. A white lung sign (Figure 4B) occurs when there are too many vertical lines in one zone, which indicates greater severity of disease. When treatment is effective, a decrease in vertical line density is observed. In summary, to differentiate between ARDS and pulmonary edema, the pleural line will be thicker (>3 mm) and irregular with subpleural consolidations and skip areas of normal tissue in ARDS, whereas pulmonary edema is identified by the pleural line being thinner (<2 mm) and regular with no subpleural consolidations and no skip areas [23].

When pulmonary edema is being treated, LUS can be helpful for estimating the pulmonary artery occlusion pressure (PAOP). In the critically ill, the PAOP can be estimated by examining the presence of horizontal lines (A lines) and the vertical lines (B Lines) [24]. The predominance of horizontal A lines has >90% specificity that the PAOP is <13 mm Hg, whereas a predominance of vertical B lines is indicative of interstitial syndrome and associated with different ranges of PAOP, with no conclusive results. Vertical lines are usually closer to each other and about 3 mm apart at the level of the pleural line in patients with pulmonary edema, while in patients with ILD or ARDS, the separation is about 7 mm [2]. Maw et al. [25] conducted a meta-analysis studying the accuracy of LUS in the diagnosis of pulmonary edema in patients with heart failure compared with the accuracy of chest X-rays, and they found that LUS had a sensitivity and specificity of 88% and 90%, respectively, compared with chest X-rays, which had a sensitivity and specificity of 73% and 90%, respectively.

Subpleural micro or macro consolidation signs are usually associated with pulmonary fibrosis, ARDS, or pneumonia. Subpleural consolidations may be micro or macro consolidations and lead to interruptions of the continuity of pleural line [4,26]. These consolidations should be noted and described when performing LUS, and the location should be identified because it can correlate with the disease process. The consolidations are expected to resolve with treatment in cases such as pneumonia, and LUS can help monitor the progression of disease and treatment effect [27].

Shred signs (Figure 4C) are limited and small, and owing to their irregular borders, they resemble a disturbed tissue pattern below the pleural line. Shred signs represent a non-trans-lobar consolidation, and the border between the consolidated and aerated lung is irregular as well. These signs are usually associated with pneumonia. In a prospective study with pneumonia confirmed by clinical diagnosis and computed tomography scan of the chest, the subpleural consolidations identified by LUS had a sensitivity of 90% and a specificity of 98% [28]. In a systematic review and meta-analysis, Chavez et al. [29] found similar results, with LUS having a sensitivity of 94% and a specificity of 96% for the diagnosis of pneumonia. In the context of COVID-19, most of the mentioned signs have been found to be present, including the micro consolidation, with interruptions of the pleural line, skip areas, and multiple vertical B lines [30]. In addition to pneumonia, the shred sign can also be present in patients with pulmonary embolism (with high sensitivity over 90% if combined with echocardiography and vascular lower extremity ultrasound) [31].

The tissue-like pattern sign (Figure 4D) occurs in more severe cases, with the consolidation being larger and the lung tissue ultrasound images resembling those of liver parenchyma. This sign usually represents trans-lobar consolidation with complete loss of aeration in the region, and it has high sensitivity and specificity, 93% and 98%, respectively [32]. When the consolidation is large, rib shadows limit a complete view, and it is necessary to use a horizontal orientation of the transducer, with the transducer face being situated fully between two ribs. The new guidelines also suggest the use of contrast-enhanced LUS evaluations whenever possible to obtain further information about peripheral consolidations [4]. When contrast is used, the MI should be set low owing to the risk of developing pulmonary capillary hemorrhage secondary to microbubble air rupture. MI should be between 0.4 and 0.7, and prolonged use and high-output Doppler should be avoided [33].

Signs Originating From the Lung Parenchyma

Patients with pneumonia can demonstrate an air bronchogram sign within the tissue-like pattern of the lung. This area of hyperechoic shadows, which are usually longitudinal but could be punctate dotlike figures, represents trapped air within a bronchus. A dynamic air bronchogram (Figure 4E) will show movements of the hyperechoic line in the bronchioles surrounded by the lung parenchyma with respiration [34,35]. This observation usually favors the presence of infectious process and pneumonia [36,37]. For cases of suspected pneumonia, the new guidelines recommend using contrast-enhanced LUS whenever possible to obtain further information about the consolidation identified. This approach can be used for patients with pleural-based lesions. A small amount of contrast, such as sulfur-hexachloride microbubble contrast media, can be given intravenously, and the time to enhancement and extent of enhancement of the lesions can be examined [33]. Using contrast enhancement can help differentiate a pneumonic process from pulmonary embolus or atelectasis based on the different time and extent of contrast enhancement. As previously mentioned, when contrast is used, the MI should be set between 0.4 and 0.7 to avoid tissue damage [33]. Dynamic air bronchogram is shown in Video 3.

**Video 3:** Dynamic air bronchogram.

When an air bronchogram is noted but shows no movement with respiration (static air bronchogram; Figure 4F), it indicates a possible atelectasis rather than lobar pneumonia. Static air bronchogram is shown in Video 4.

**Video 4:** Static air bronchogram.

Buda et al. [35] studied micro-atelectasis in patients who were intubated on positive pressure ventilation. The use of LUS aided in the diagnosis and management of micro-atelectasis near the pleural surface that was caused by intubation and mechanical ventilation in patients, with observations based on monitoring the micro-consolidation that developed after intubation. With the adjustment of positive end expiratory pressure, the simultaneous LUS use enabled correctly identifying the effect of recruitment when pressure was increased. Table 1 summarizes the clinical significance of the different LUS signs and artifacts. Pleural, subpleural, and parenchymal signs are shown in Figure 4.

The aeration score (LUS score) is calculated based on a pattern of increasing vertical B lines and the loss of the horizontal A line pattern; that is, a decreasing air/tissue ratio indicates a progressive homogeneous loss of aeration and development of increasing hypoxemia. As the vertical B lines increase in number and increasingly coalesce, a more severe disease process is indicated [5,38]. When a complete loss of aeration occurs, LUS will demonstrate a tissue-like pattern [39,40]. The aeration score is used to identify the severity of lung disease on a numerical scale, and it is calculated by adding the number of vertical B lines in each of the six zones on each side. Each zone is scored as follows: 0 = no more than two vertical lines; 1 = three separate vertical lines; 2 = coalescent vertical lines; and 3 = tissue-like pattern. The total score can range from 0 to 36 for both lungs combined. A similar score was validated by a recent study on COVID-19 [41,42] and in a meta-analysis on ARDS patients in which the investigators examined lung zones and scoring systems [41-43].

Artificial intelligence (AI)

Newer generations of ultrasound machines have AI capability, and the integration of this technology enables the identification of many of the LUS signs and artifacts, including the vertical B lines, which can be used to quickly and repeatedly monitor a patient’s response to treatment. Arntfield et al. [44] demonstrated the ability of deep AI machine learning to correlate and identify the vertical B lines and horizontal A lines with a sensitivity and specificity of 90% and 92%, respectively, compared with individual manual analysis. Roshankhah et al. [45] investigated the training and testing for automated segmentation of lung areas and the ability of the ultrasound machine to identify vertical lines, their coalescence, and aeration scores, finding an average correlation coefficient of 0.89. Some ultrasound machines have the capability and software to combine multiple images of different zones into one larger image. When the operator performs a simple sweep (slide the transducer across multiple lung zones), a combined image of the different zones of lung parenchyma and the pleural line can be generated [46]. This ability can reduce the need to start and end at each zone and integrates all images in one sweep. These early studies on AI show that it can help providers perform LUS exams more frequently, with a shorter time needed for analysis and more standardized results achieved.

Pleural effusions

Pleural Effusion Diagnosis by Ultrasound

Evaluation of pleural effusions in ICU patients is most often done when patients are in a supine position. Initially, the lower lateral zone between the anterior and posterior axillary lines is examined for the presence of fluid. When effusion is absent, the curtain sign [47] will be present, which represents the diaphragm and the subdiaphragmatic structures becoming covered by the air-filled lung moving downwards during the inspiratory phase. Curtain sign shown in Video 5.

**Video 5:** Curtain sign.

**Figure 5:**
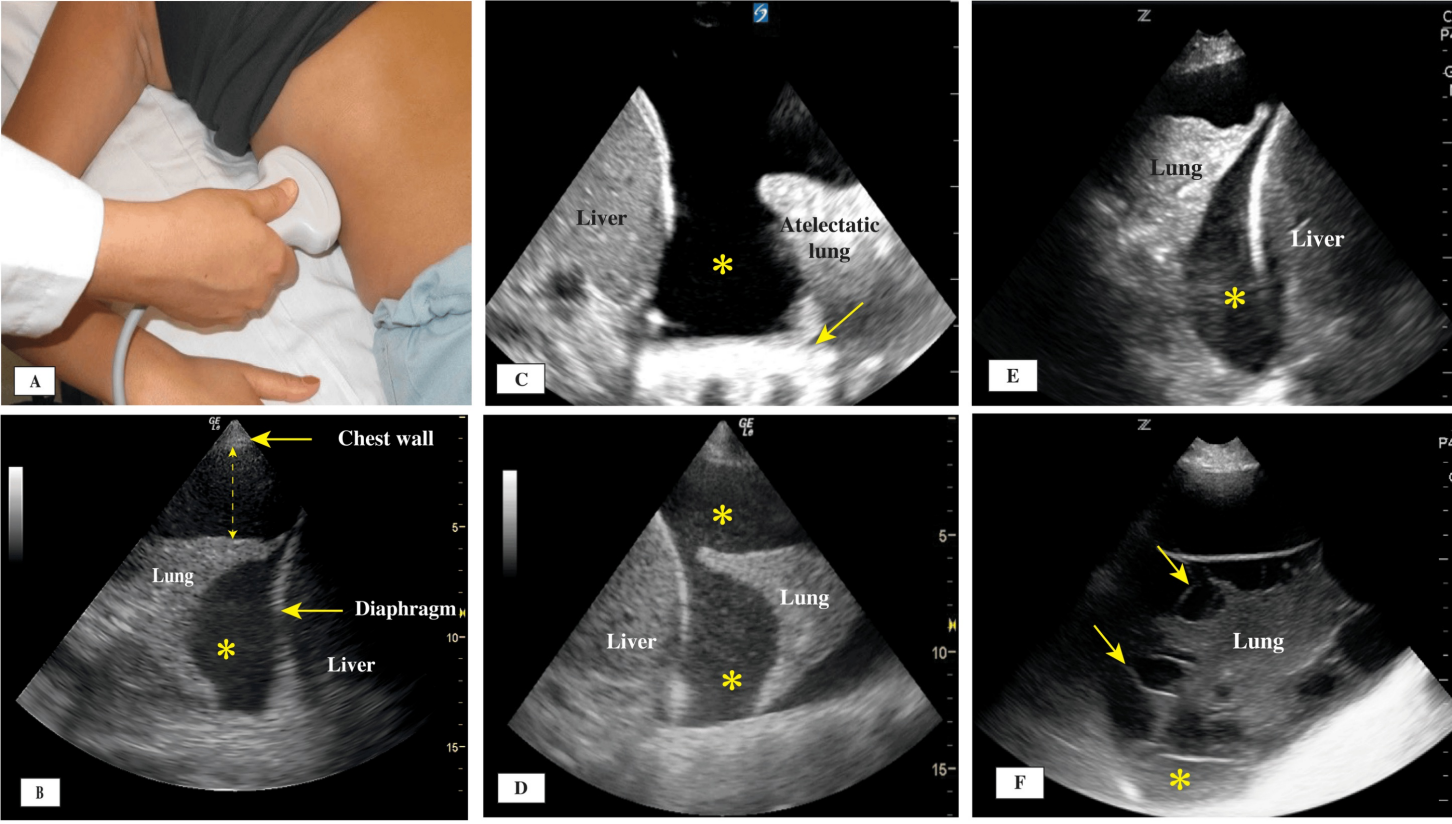
(A) Posterolateral point area for transducer placement. (B) Pleural effusion with jellyfish sign. The dashed arrow measures the distance between the lung and chest wall for estimation of the pleural effusion size. (C) Simple anechoic pleural fluid (asterisk) and the thoracic spine sign (arrow). (D) Complex echoic pleural fluid with floating debris (plankton sign) (asterisks). (E) Complex echoic pleural fluid with debris accumulating in the dependent area owing to hemothorax (hematocrit sign; asterisks). (F) Complex echoic pleural fluid with septations (arrow) and thick debris accumulating in the dependent area (empyema; asterisk).

For a supine patient, placing the transducer in the posterior-lateral point or area (zone 3) is recommended (Figure 5A). Usually, the fluid will appear as an anechoic space occupying the area between the lungs, chest wall, and diaphragm. The thoracic spine can be identified easily when fluid is present (the thoracic spine sign [48]), and the vertebral bodies above the diaphragm will be visible (Figure 5C). The jellyfish sign [49] is a common finding when pleural effusion is moderate to large, and it represents the lung movement within the fluid-filled space with respiration as shown in Video 6.

**Video 6:** Jellyfish sign.

**Figure 6:**
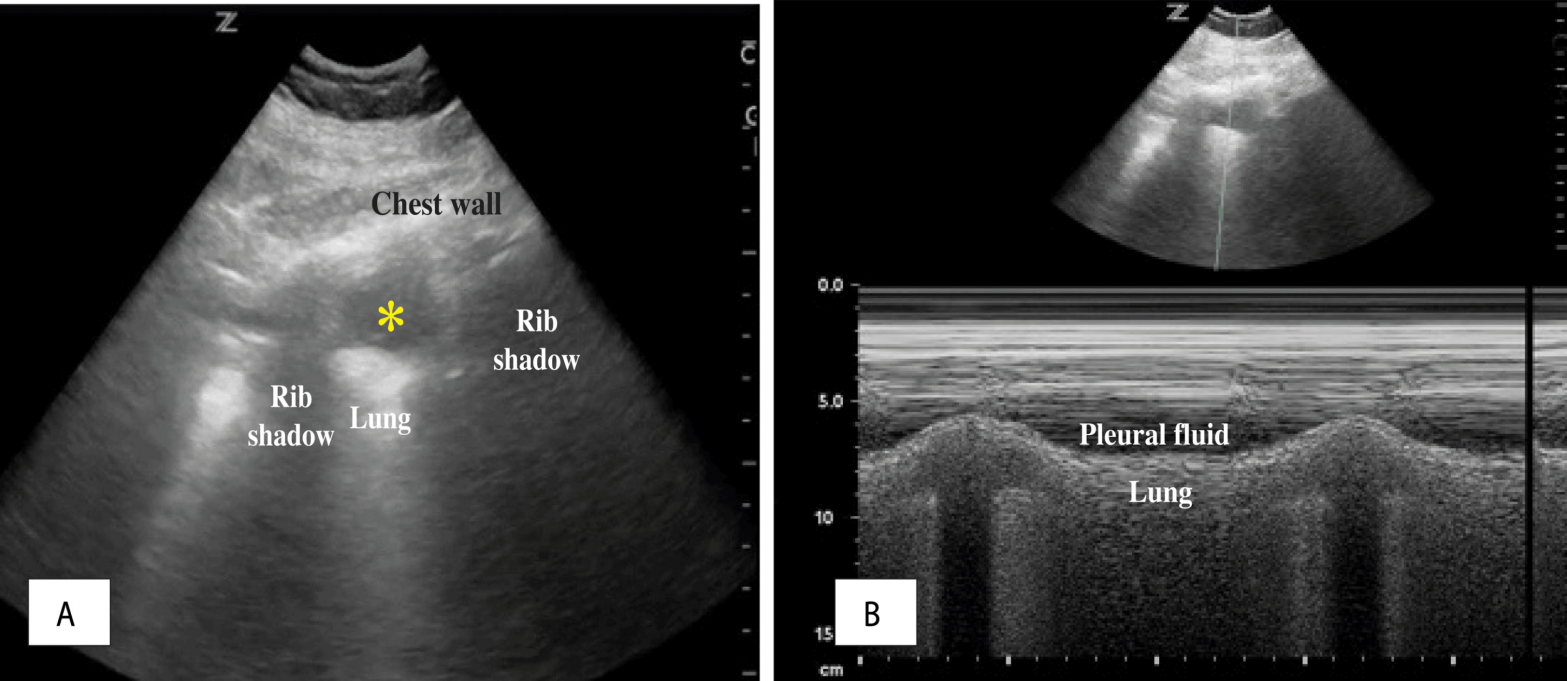
(A) Quad sign formed between the chest wall superiorly, pleural line inferiorly, and rib shadows on sides (asterisk); (B) M-mode of the quad sign showing the sinusoid sign.

The quad sign [50], which is specific for pleural effusion, is defined by the pleural fluid being delineated by the pleural line (upper border), lung line (visceral pleural, i.e., lower border), and the two rib shadows on the sides (Figure 6A). Applying the M-mode to the quad sign area will result in what is called the sinusoid sign (Figure 6B) [40], which represents the movement of the lung (atelectatic mostly) toward the chest wall during the respiratory cycle. Examples of pleural fluid examinations are presented in Figure 5. Pleural fluid with quad and sinusoid signs is shown in Figure 6.

Pleural Effusion Characteristics

The main ultrasound characteristics of pleural fluid of different etiologies are listed in Table 2 [51-57], and can be divided in general into the following categories: (1) simple anechoic fluid, which is usually transudative in nature with a negative predictive value of 90%; (2) complex echogenic effusions represented by heterogenous echogenic material present inside the anechoic space that are usually indicative of exudative nature with a positive predictive value (PPV) of 90%; and (3) complex echogenic homogenous or complex echogenic with septate effusions, which are usually indicative of exudative nature with a PPV of 96% [51].

**Table 2:** Pleural line and effusions characteristics by ultrasound. PPV: positive predictive value

Disease	Pleural line	Pleural fluid	Diaphragm
Heart failure, renal failure, cirrhosis	Thin (<2 mm), regular, no skip areas, no nodules	Anechoic fluid tending to reaccumulate more on the right than the left. No debris unless chronic with organizing inflammatory process.	Usually no dysfunction.
Blood	Thin (<2 mm), regular, no skip areas, no nodules	Anechoic/echoic. Mostly, there is echogenic fluid that tends to accumulate in the gravity-dependent area (hematocrit sign). A combination of swirling echogenic debris (plankton sign) and hematocrit sign is highly suggestive of hemothorax.	No dysfunction.
Empyema	Thin, becomes thick in later stages; increases irregularity with chronicity; skips areas	The appearance depends on the composition of the collection. There will be some non-uniformed echogenicity and septations. There are three stages: (1) exudative stage with floating debris that is gravity dependent; (2) fibrinous stage in which fibrin and septations develop, especially between the atelectatic lung and the diaphragm; and (3) organized stage, in which a mass-like structure forms with thickening of the pleural line.	Diaphragmatic function could be limited by adhesions.
Malignant fluid	Increase thickness; irregular; nodules present; pleural thickness >10 mm + irregularity + nodules = malignancy with PPV 100%; subpleural consolidations with static or dynamic air bronchogram (about 50% of cases)	Nonspecific. Could be anechoic, but it will mostly develop echogenicity with chronicity and infections, and debris will form.	Diaphragmatic paralysis occurs in about 30% of cases.
Inflammatory or connective tissue disease	Could be thickened with chronic disease (>2 mm), irregular, skip areas, nodules present	Pleural fluid is usually anechoic. Associated with echogenicity as the disease progresses and becomes chronic. Pleural nodularity will be present.	Dysfunctional diaphragm.

In general, transudative fluids are anechoic, while exudative fluids can be anechoic but will mostly have floating debris and fibrinous stranding with septations and loculations in chronic disease such as complicated empyema (Figures 5A-5F).

Pleural fluid pixel density has been studied and can also be used to characterize the nature of the fluid. The density is higher in exudative fluid compared with transudative fluid, averaging 6.65 versus 2.74 pixel density, respectively [54].

In cases in which cellular debris is present in the pleural fluid secondary to blood, the floating and swirling debris causes an increase in echogenicity, which is called the plankton sign (Figure 5D) [2]. If the debris accumulates more in a gravity-dependent area, it is called the hematocrit sign (Figure 5E) [56]. Acutely accumulated fluid usually does not have septations, but when septations develop, it could indicate an infectious etiology as well as chronicity of the disease process. Malignant fluids vary significantly and could present as clear fluid in the acute phases, with progression to complex fluid with adhesions and septations in chronic cases, owing to complications from repeated infections and empyema development. Malignant conditions can also lead to pleural thickening, with irregularity and nodularity of the pleural surface and diaphragmatic thickening of more than 1 cm. Pleural thickening with correlated clinical findings could be specific for the presence of malignancy with 88% sensitivity and 48% specificity [54,56]. Differentiation between pleural fluid of different origins is presented in Table 2.

Pleural Effusion Volume Estimation

In the ICU, assessments are done to identify whether a small, moderate, or large pleural fluid collection is present. Studies estimating the volume have different results depending on the patient’s position, with volume estimation by ultrasound being relatively more accurate when the patient is in a sitting position than in a supine position [58]. Many ultrasound methods have been proposed to estimate the pleural effusion volume [59]. For a more practical approach in the ICU, the Balik formula [60], which has a moderate correlation of 0.52, is used. The patient is placed in a recombinant position of 15°, the transducer is placed in the posterior lateral aspect and perpendicular to the chest wall with the indicator pointing upwards, and the interpleural distance is measured at end inspiration (Figure 5B). This distance is multiplied by 20 to estimate the volume as shown in the formula:

$\text{Effusion volume (in milliliters)} = \text{Distance between visceral and parietal pleura (in millimeters)} \times 20$

Eibenberger et al. [61] estimated the pleural effusion size in patients in supine position. The transducer was placed in the posterior lateral aspect and perpendicular to the chest wall, with the indicator pointing upwards towards the ceiling. The distance between the chest wall and lung was then measured at end inspiration. This study showed a better correlation at 0.8; however, this formula has limited value if the effusion thickness is <10 mm:

$\text{Effusion volume (in milliliters)} = 47.6 \times \text{Distance between visceral and parietal pleura} - 837$

Diaphragm

**Figure 7:**
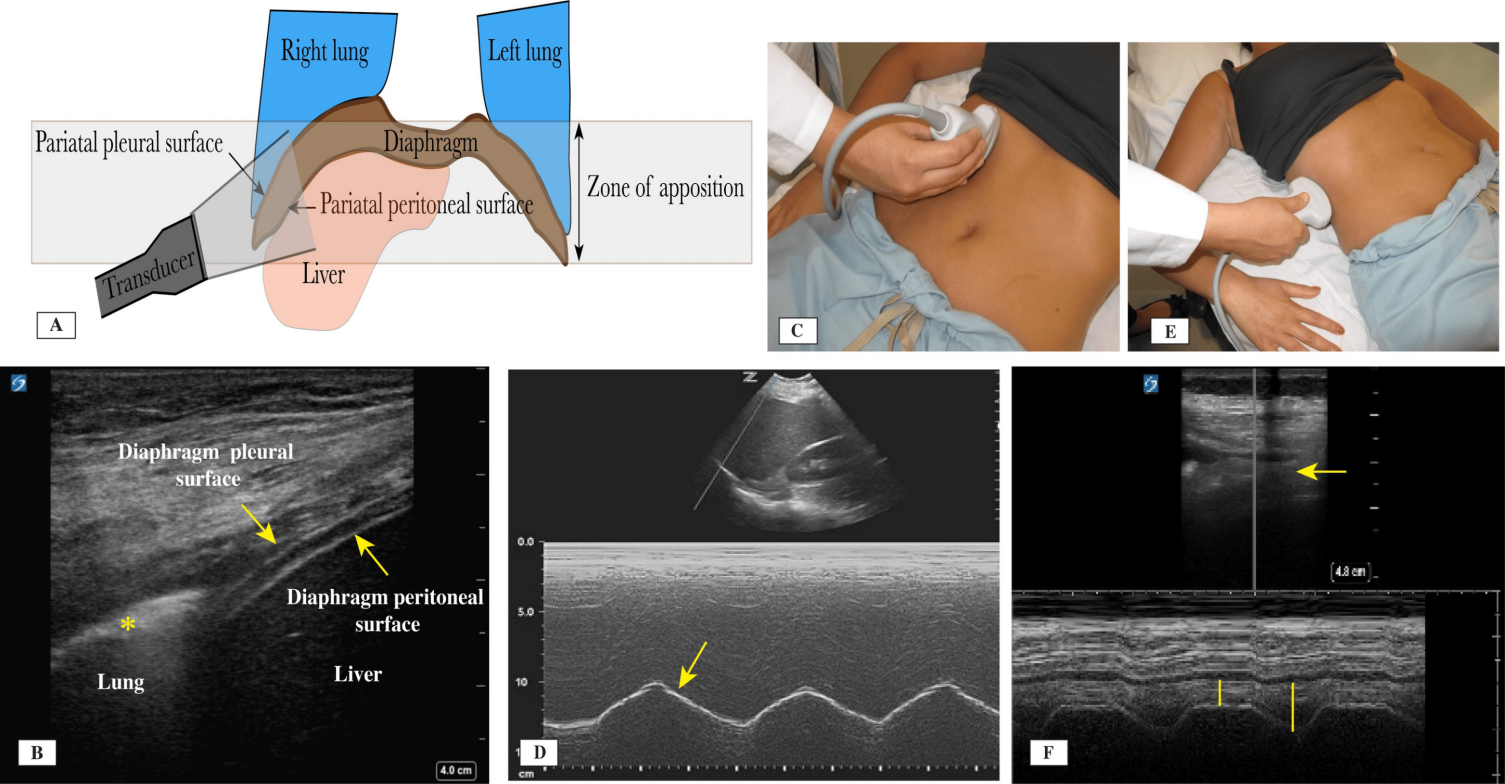
(A) Illustration of the zone of apposition with the peritoneal and pleural surfaces. (B) Zone of apposition showing the pleural line (asterisk) reaching the diaphragm. (C) Diaphragm excursion measurement transducer placement. (D) M-mode showing the amplitude of the diaphragmatic excursion. (E) Transducer position and orientation to measure the diaphragmatic thickness. (F) M-mode of the diaphragm thickness changes with the respiratory cycle (yellow bands). Credit: Image A created by the authors.

Assessment of the diaphragmatic function is being done more frequently in ICU patients. Diaphragmatic dysfunction can result from prolonged mechanical ventilation, trauma, sepsis, and other causes [62]. Assessment can identify diaphragmatic failure when weaning patients from mechanical ventilation fails [63]. It is also used in combination with other parameters for lung function to identify hyperinflation in patients with COPD [64]. The process includes measurements of the excursion and thickness changes of the diaphragm during the respiratory cycle. The zone of apposition is where the diaphragm joins with the pleural surfaces, and it gives the operator a good idea about the structure and function of the diaphragm (Figures 7A-7B and Video 7). The zone of apposition with diaphragm examination sites is illustrated in Figure 7.

**Video 7:** Normal diaphragmatic function at the zone of apposition.

Diaphragmatic muscle excursion is better evaluated with the use of a phased array or curvilinear transducer placed between the mid-clavicular and the anterior axillary line in the subcostal region (Figure 7C). Inspiratory amplitude and excursion in a semi-recumbent patient with normal tidal breathing averages >14 mm and in a supine patient >11 mm. Diaphragmatic weakness is diagnosed with tidal excursion <10-15 mm [65]. Using M-mode helps to achieve more accurate measurements of the excursion (Figure 7D).

Diaphragmatic thickness and its changes are measured during the respiratory cycle using a linear transducer placed at the zone of apposition at about the 8th to 10th intercostal space mid-axillary line, 0.5-2 cm below the costophrenic angle (Figure 7E). At this position, the three-layered structure at the zone of apposition is observed, consisting of the pleural and peritoneal hyper-echoic membranes and a hypoechoic diaphragmatic muscle in between (Figure 7B). During inspiration, an increase in the diaphragmatic thickness of about 20% or more is normally expected. Calculating the thickening fraction of diaphragm (TFdi) change can be done using the following formula:

$\text{TFdi} = \left( \frac{\text{Thickness in inspiration} - \text{Thickness in expiration}}{\text{Thickness in expiration}} \right) \times 100$

When performed in a mechanically ventilated patient, TFdi >30-36% has been shown to predict successful liberation, while diaphragm weakness is diagnosed when TFdi (max) <20% (Figure 7F) [65-67]. Parada-Gereda et al. [68] conducted a systematic review and meta-analysis on the usefulness of diaphragmatic ultrasound in predicting the success of liberation from mechanical ventilation and found that diaphragm excursion has a sensitivity and specificity of 80% and diaphragmatic thickening fraction has a sensitivity of 85% and a specificity of 75%. In the future, less heterogeneous studies are needed for inclusion in meta-analyses to form a full consensus regarding the cutoff numbers for the diaphragmatic function. Normal quiet respiration with diaphragmatic movement is shown in Video 7.

Chest ultrasound and procedures

Ultrasound is used to help in many procedures in the ICU, including pleural fluid drainage, pleural catheter insertion, and percutaneous tracheostomy [69]. In this section, the practical aspects of implementing ultrasound in these procedures are discussed.

Thoracentesis

**Figure 8:**
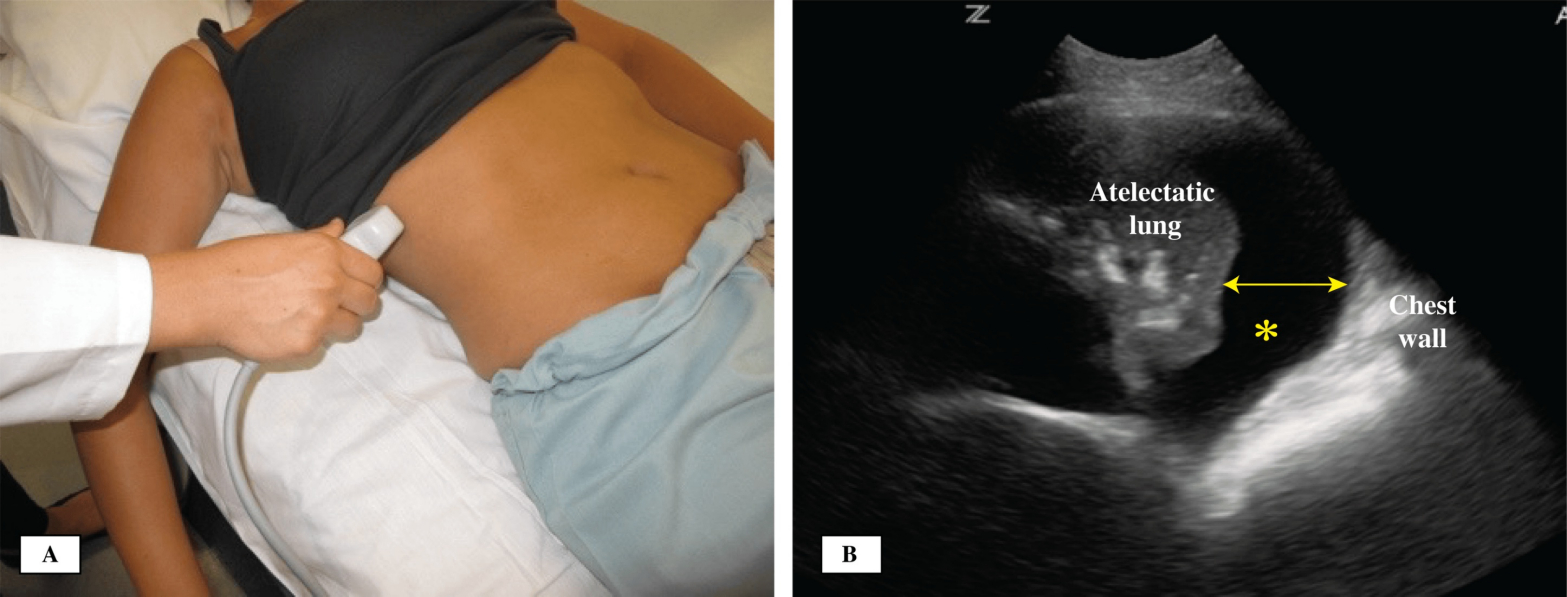
(A) Transducer placement in the posterior lateral zone to estimate pleural effusion size; (B) Pleural fluid size measurement with the widest diameter. Clear pleural fluid is marked with an asterisk.

Performing thoracentesis without the use of ultrasound is usually discouraged owing to the unacceptably high pneumothorax complications of 20-39% [70,71]. Many societies’ recommendations, including the British Thoracic Society Pleural Disease Guidelines, advise using ultrasound when placing catheters for pleural fluid drainage [72]. Ultrasound can help in identifying the optimal puncture site and determining the depth of needle insertion to reach the parietal pleural line [73]. Techniques are mostly static by identifying the best site for insertion, marking the site, identifying the trajectory for the needle path, and carrying out the procedure shortly after. The dynamic technique is less commonly used and difficult to perform if the procedure is carried out by one operator. The most reliable site is the safe triangle that lies between the lateral edge of the pectoralis major superiorly, the lateral edge of the latissimus dorsi inferiorly, the base of the axilla cephalad, and the fifth intercostal space caudal. To perform the procedure, it is recommended to find the largest pocket of fluid closest to the chest wall (Figure 8) [74]. Color and pulse-wave Dopplers can be utilized to identify subcostal blood vessels prior to chest instrumentation [75], especially in patients at high bleeding risk. Examination of the pleural fluid size is illustrated in Figure 8.

Tracheostomy and Cricothyrotomy

**Figure 9:**
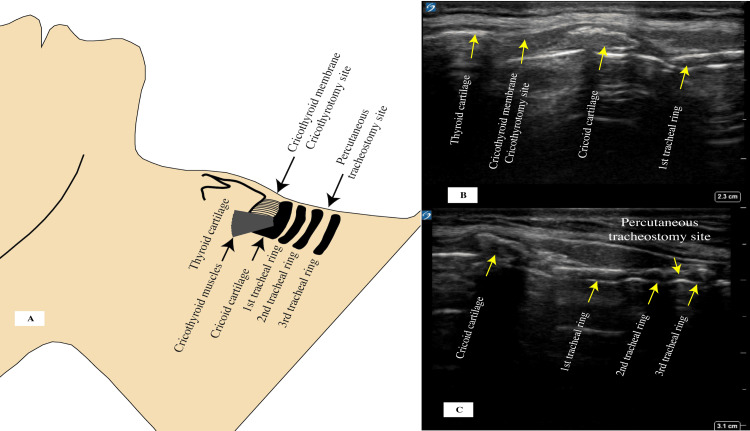
(A) Cricothyroid membrane and anatomy for cricothyrotomy and percutaneous tracheostomy. (B) Ultrasound image showing the relation of the cricothyroid membrane to the thyroid and cricothyroid cartilages with the first and second tracheal rings. (C) Cricothyroid cartilage with the sub cricoid space and the first three tracheal rings. Credit: Image A created by the authors.

Ultrasound can be used as a screening tool to identify anatomical variations and aberrant blood vessels when performing a tracheostomy or during an emergent cricothyrotomy. In an emergent airway procedure, such as a cricothyrotomy, ultrasound can be very helpful in determining the optimal puncture site because it enables the operator to identify the thyroid gland, thyroid cartilage, cricothyroid membrane, cricoid cartilage, and the tracheal rings (Figure 9). The incision is done along the long axis of the cricothyroid membrane, and a small cricothyrotomy tube is placed, which can be done using the Seldinger technique. Identifying blood vessels is also crucial prior to performing the procedure, and the use of ultrasound can also help estimate the distance between the skin and trachea [76], enabling the operator to avoid puncture of the posterior wall by the needle. Bedside tracheostomy is done under more controlled settings, and the incision site is usually between the second and the third tracheal rings [77]. The addition of ultrasound guidance during tracheostomy can help identify anatomical variations and enable visualization of the needle insertion and its path with confirmation of tracheostomy tube placement by the bronchoscope as well as ultrasound [78]. Anatomy of cricothyrotomy and tracheostomy is illustrated in Figure 9.

Limitations of LUS

LUS and diaphragmatic ultrasound have limitations, and it is imperative to combine the clinical scenario with the ultrasound findings to reach the best diagnosis. One of the well-recognized signs is lung sliding, the absence of which is a hallmark feature in pneumothorax identification. There are limitations to lung findings on lung sliding, where other conditions such as ARDS, pneumonia, atelectasis, airway obstruction, and ILD can also lead to a lack of lung sliding by mechanisms such as adhesion formation.

Establishing the volume of pneumothorax by LUS can be challenging because the operator will need to identify and locate the lung point to be able to estimate the pneumothorax size. A lung point can be difficult to find on many patients, and in cases of very large pneumothorax, it may not be possible to locate the lung point [79].

LUS currently has a limited role in the diagnosis of severe emphysema and bullae in patients with severe disease without heavy reliance on the clinical scenario. Except for the hyperresonance and predominance of the horizontal lines (A lines), signs or artifacts to help identify patients with COPD are limited.

Although LUS is very helpful in detecting pneumonia, it typically does not aid in identifying the cause. In one study focused on the H1N1 influenza pandemic of 2009, children with the viral infection were found to have smaller sub-pleural consolidations than children with bacterial pneumonia [80]. This finding has not been widely validated, and even during the COVID-19 pandemic, the difference in size of the sub-pleural consolidation was not clear in the majority of studies. An open question was whether the rapid progression of the disease led to greater damage to the lungs and the development of ARDS, which could have led to a pattern similar to that of severe bacterial pneumonia [81]. As previously mentioned, the clinical picture and findings of other diagnostic modalities can help narrow the differential diagnosis and identify the cause of pneumonia.

The practical aspect of LUS is also challenging, including the availability of dependable high-quality ultrasound machines, with cost being a major limiting factor for many institutions and providers. Despite an increase in LUS training in the past few years, this technology has not been fully incorporated into medical schools’ curricula. An increasing number of medical schools have started to adopt LUS education during the past 15 years [82], but more needs to be done. Finally, performing LUS requires a provider’s time, and in many instances, having to do multiple examinations in a single day could be a barrier for busy providers with no clear reimbursements.

## Conclusions

In this review, we aimed to focus on the clinical and practical aspects of LUS, emphasizing the new guidelines. LUS applications are still expanding, and with the advancement and introduction of new technologies, the ability to use LUS at bedside will increase. This development will help clinicians answer questions in real time and improve management of patients.
